# Elucidating the Mechanisms of Acquired Palbociclib Resistance via Comprehensive Metabolomics Profiling

**DOI:** 10.3390/cimb47010024

**Published:** 2025-01-02

**Authors:** Lulu Yang, Yajun Yue, Zhendong Wang, You Jiang, Zhichao Xue, Yongzhuo Zhang

**Affiliations:** 1 Technology Innovation Center of Mass Spectrometry for State Market Regulation, Center for Advanced Measurement Science, National Institute of Metrology, Beijing 100029, China; yangluluaxx@163.com (L.Y.); jiangyou@nim.ac.cn (Y.J.); 2 General Management Department of Laboratory Base, National Institute of Metrology, Beijing 100029, China; yueyj@nim.ac.cn; 3 Center for Advanced Measurement Science, National Institute of Metrology, Beijing 100029, China; 232086001021@lut.edu.cn (Z.W.); zhangyz@nim.ac.cn (Y.Z.)

**Keywords:** LC-MS, SW620 cells, metabolomics, drug resistance, cancer biology

## Abstract

Palbociclib is a cyclin-dependent kinase 4/6 inhibitor and a commonly used antitumor drug. Many cancers are susceptible to palbociclib resistance, however, the underlying metabolism mechanism and extent of resistance to palbociclib are unknown. In this study, LC-MS metabolomics was used to investigate the metabolite changes of colorectal cancer SW620 cells that were resistant to palbociclib. The study indicated that there were 76 metabolite expression differences between SW620 cells with palbociclib resistance and the parental SW620 cells involving amino acids, glutathione, ABC transporters, and so on. MetaboAnalyst 6.0 metabolic pathway analysis showed that arginine synthesis, β-alanine metabolism, and purine metabolism were disrupted. These results may provide potential clues to the metabolism mechanism of drug resistance in cancer cells that are resistant to palbociclib. Our study has the potential to contribute to the study of anti-palbociclib resistance.

## 1. Introduction

Cancer, a highly heterogeneous disease, manifests in a broad spectrum of signs and symptoms, astutely evading the immune system while harboring its own intricate aberrations, many of which remain undetected by the immune system [1]. Moreover, the metastatic potential of cancer cells accounts for the mortality of most cancer patients [2]. Despite remarkable advancements in cancer therapy that have significantly bolstered survival rates, cancer continues to hold the dubious distinction of being the foremost cause of mortality, annually claiming millions of lives. Tumor cells exhibit extreme flexibility in reprogramming metabolism to support tumor development, metastasis, and resistance to treatment. However, this also provides potential sources for therapeutic targets and biomarkers in tumor treatment [3,4]. In the study of early colorectal cancer, researchers have discovered through the metabolomic analysis of plasma that lipid metabolism and phenylacetylglutamine have potential roles in the onset and progression of early colorectal cancer [5]. While clinical interventions encompassing surgery, chemotherapy, and radiotherapy offer viable treatment options, they are often accompanied by limitations, toxicity, and the emergence of drug resistance [6]. Consequently, it is paramount to delve deeper into the mechanisms underlying cancer drug resistance and develop strategies to mitigate the adverse effects of increasing drug doses.

Palbociclib is a cyclin-dependent kinase 4/6 inhibitor, and this drug exerts antitumor effects by regulating the cell cycle [7]. Numerous studies have shown that palbociclib has been used in the treatment of a variety of cancers with remarkable efficacy [8,9,10,11,12,13]. However, tumor cells can also become resistant to palbociclib. The P21 protein is an important cell cycle regulatory protein involved in the process of cell growth, differentiation, senescence, and death, which is closely related to tumor development. It was found that breast cancer cells chronically exposed to palbociclib had increased intracellular expression of p21, which led to acquired drug resistance [14]. In colorectal cancer treatment, drug resistance is one of the major barriers to treatment, and studies have found that chemoresistance in colorectal cancer may be associated with miRNAs [15] and cricRNAs [16]. Previous studies have explored chemoresistance in cancer, but this is far from sufficient in cancer research, and the potential mechanisms of resistance to target drugs, especially palbociclib in colorectal cancer, are not yet known.

Metabolomics uses advanced analytical techniques to measure a large number of small analytic metabolites in cells, tissues, and biological fluids to characterize and refine the perturbations of the biological pathways of creatures [17,18,19]. The metabolome can serve as a powerful tool to provide information on tumor-specific metabolism [20,21,22,23]. Liquid chromatography-mass spectrometry (LC-MS) technology usually involves LC and MS coupled with electrospray ionization (ESI), atmospheric pressure chemical ionization (APCI), or atmospheric pressure photoionization (APPI), where the liquid chromatography serves as the separation system and the mass spectrometry as the detection system [24]. When LC and MS work together, multi-stage MS can be performed to deduce the structure of compounds, thus enabling more accurate qualitative and quantitative analysis [25]. LC-MS reflects the complementary strengths of chromatography and mass spectrometry, integrating the high separation capacity of chromatography for complex samples with the advantages of mass spectrometry including high selectivity, sensitivity, and the ability to provide relative molecular mass and structural information. This technology, with its superior sensitivity and specificity, is increasingly used in many areas of drug and food analyses [26,27]. Currently, the underlying mechanism of resistance to palbociclib in colorectal cancer remains poorly understood, necessitating further metabolic studies. In this study, we harnessed liquid chromatography-mass spectrometry (LC-MS) metabolomics to delve into the mechanism of palbociclib resistance in colorectal cancer.

## 2. Materials and Methods

### 2.1. Cell Culture and Reagents

SW620 cells obtained from BNCC (#337664) were cultured in a 37 °C incubator with a 5% CO_2_ supply. The culture medium consisted of 90% RPMI-I640 (Solarblo, #31800) and 10% FBS (Every Green, #11011-8611). Palbociclib (Selleck Chemicals, #S1116) was dissolved in distilled H_2_O at a stock concentration of 10 mM and further diluted to a working concentration with the cell culture medium. SW620 palbociclib resistance cells (SW620 PD_R or resistant cells) were established by slowly increasing palbociclib in the culture medium for over 6 months.

### 2.2. Cell Viability Assay

A total of 3000 cells of SW620/SW620 PD_R were seeded in 96-well plates overnight before drug treatment. The CCK8 (Cell Counting Kit-8, Biorigin, São Paulo, Brazil, # BN15201) assay was used for the cell viability measurements at the end of the drug treatments. CCK8 solution was added into the wells 1:10 *v*/*v*, and then the plates were incubated for 1.5 h at 37 °C, the optical density was measured at 490 nm, and the OD490 value was the corresponding viability value. Growth inhibition in each well was calculated as (viability control − viability drug)/viability control × 100%.

### 2.3. Pre-Treatment

SW620 PD_R and SW620 cells that were treated once with 1 μM palbociclib were set as study groups, while SW620 cells without any treatment were used as the control group. Cells were collected when the cell density reached 80%. For sample preparation, the cells were first rinsed with PBS twice and then with ammonium formate solution at a concentration of 150 mM. Afterward, 1.2 mL of 50% MeOH was added into each well for the extraction of intracellular metabolites. Samples were completely lysed using an ultrasonic homogenizer in an ice bath for 30 min and centrifuged at 14,000 rpm under 4 °C for 5 min. The supernatant was collected at overnight and stored at −80 °C before LC-MS analysis.

### 2.4. LC-MS Analysis

The supernatants were analyzed by LC-MS simultaneously after centrifugation. A total of 100 μL of the supernatant was transferred to a new vial and analyzed by a Thermo U3000-Orbitrap Elite LC-MS system (Waltham, MA, USA) equipped with an electrospray ionization (ESI) source. Liquid chromatography was performed on a Waters XBridge BEH amide column (150 × 2.1 mm, 2.5 µm particle size, Waters Corporation, Milford, MA, USA). The mobile phase for chromatographic separation was composed of phase A: 10 mM ammonium hydroxide and 10 mM ammonium acetate in 95% H_2_O/5% ACN and phase B: 10 mM ammonium hydroxide and 10 mM ammonium acetate in 95% ACN/5% H_2_O. Elution gradients were set as: 0–1 min (90% B), 1–11 min (90–40% B), 11–15 min (40% B), and 15–17 min (40–90% B). The ionization mode was ESI, and the positive ion mode was used for scanning. A total of 10 μL of each sample was injected for positive ion electrospray ionization analysis. The data analysis was conducted by MetaboAnalyst 6.0, and the raw MS files were uploaded and analyzed automatically.

### 2.5. Bioinformatics Analysis

Statistical analysis of growth inhibition was performed by GraphPad Prism 9.0, and we used the Nonlin fit of transform X to compute the IC50. For the metabolomics data analysis, the intensity value of each metabolite in each sample was first normalized to the corresponding values pooled in the QC group. Missing value imputation (KNNmethod),volcano plot (fold change > 2, *p* value < 0.05), principal component analysis (PCA), partial least squares discriminant analysis (PLS-DA), biomarker selection, pathway analysis overview as well as heatmap clustering of the altered metabolic profiling analysis were performed using the open source online platform MetaboAnalyst 6.0. The metabolic pathways were also analyzed using “Wukong”, which is a platform based on the internally provided, free-of-charge R language (https://wkomics.omicsolution.com/wkomics/main/ (accessed on 22 July 2024)).

## 3. Results

### 3.1. The SW620 PD-R Cells Were Trained to Be Resistant

**Figure 1:**
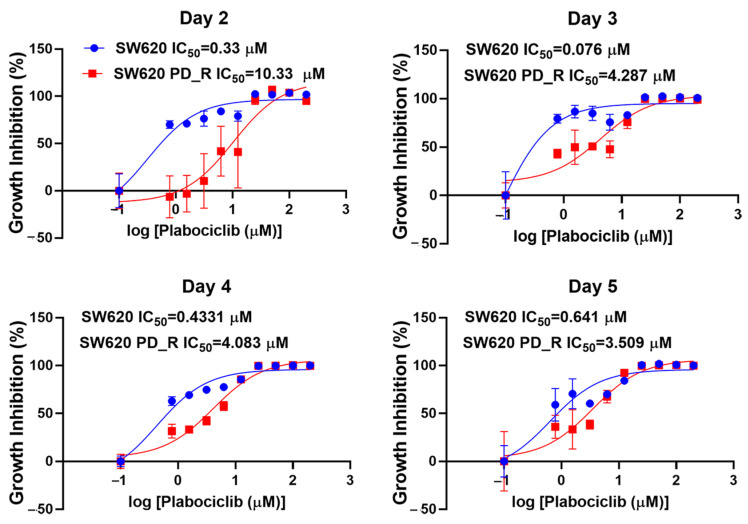
Growth inhibition of SW620 cells and resistant SW620 cells exposed to different concentrations of palbociclib for 2 days, 3 days, 4 days, and 5 days. Blue represents SW620 cells, and red represents SW620 PD_R cells.

**Figure 2:**
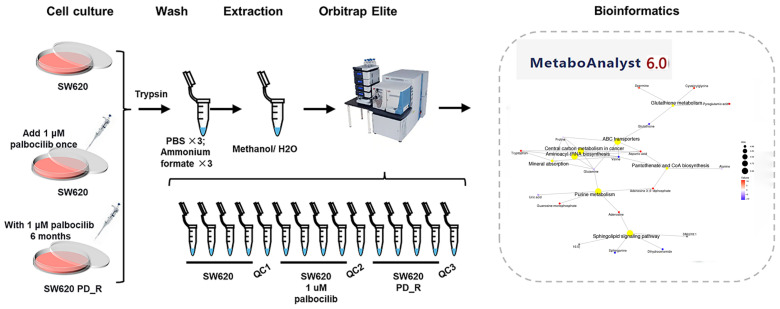
Flowchart of experimental operation. There were three experimental groups. The first experimental group was SW620 cells without any treatment; the second experimental group was SW620 cells treated once with 1 μM palbociclib; and the third experimental group was drug-resistant SW620 cells treated with 1 μM palbociclib for 6 months.

The IC50 values of the SW620 cells exposed to palbociclib for 2, 3, 4, and 5 days showed a biphasic pattern of increasing and then decreasing (Figure 1). Specifically, an IC50 value of 0.33 μM was observed after 2 days of treatment, which decreased to 0.076 μM after 3 days and increased to 0.641 μM after 5 days. Throughout the experimental period, a continuous decreasing trend in IC50 was observed in the SW620 PD_R cells. The IC50 of the SW620 PD_R cells was significantly higher than that of the SW620 cells at the four time points of 2, 3, 4, and 5 days of palbociclib treatment, which indicated the drug resistance of SW620 PD_R cells. In the subsequent experiments, we used parental SW620 cells, parental cells treated with 1 μM palbociclib, and resistant cells for further metabolomic experiments (Figure 2), aiming to elucidate the changes of metabolites in different cellular environments.

### 3.2. LC-MS Metabolic Profiling of SW620, SW620 + 1 μM PD, and SW620 PD_R Cells

#### 3.2.1. LC-MS of Metabolic Profiles

**Figure 3:**
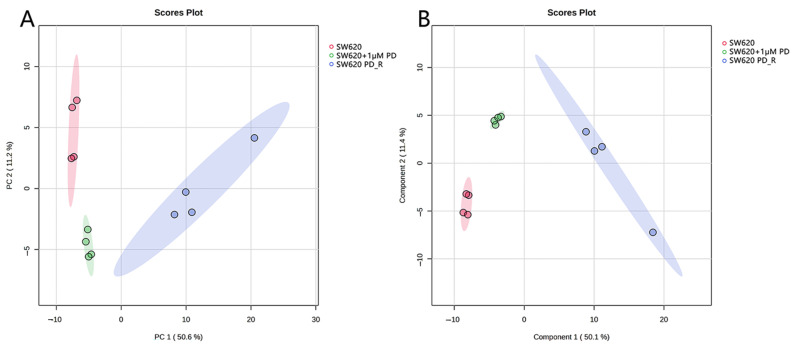
Score plots of the PCA (**A**) and PLS-DA (**B**) models for the metabolome data obtained by LC-MS showing the metabolic profile differences between the control and palbociclib-treated groups. Red cycle: control group; green cycle: SW620 + 1 μM PD group; blue cycle: SW620 PD_R group.

We found that 196 metabolites were detectable in the three groups: SW620, SW620 + 1 μM PD, and SW620 PD_R. We analyzed the metabolic profiles of the SW620 cells, SW620 + 1 μM PD cells treated with 1 μM palbociclib, and SW620 PD_R cells. Based on the LC-MS data, the PCA score plots of the metabolites showed significant separation between the three experimental groups of SW620, SW620 + 1 μM PD, and SW620 PD_R cells (Figure 3A). PLS-DA was further performed to reveal the metabolic bias between the control group and the palbociclib-treated experimental groups. As shown in Figure 3B, the metabolite profiles of the three groups had significant separation, with components 1 and 2 accounting for 11.4% and 50.1% of the total variance, respectively, in the PLS-DA model plot, indicating that palbociclib resulted in significant metabolic disturbances in the SW620 cells.

**Figure 4:**
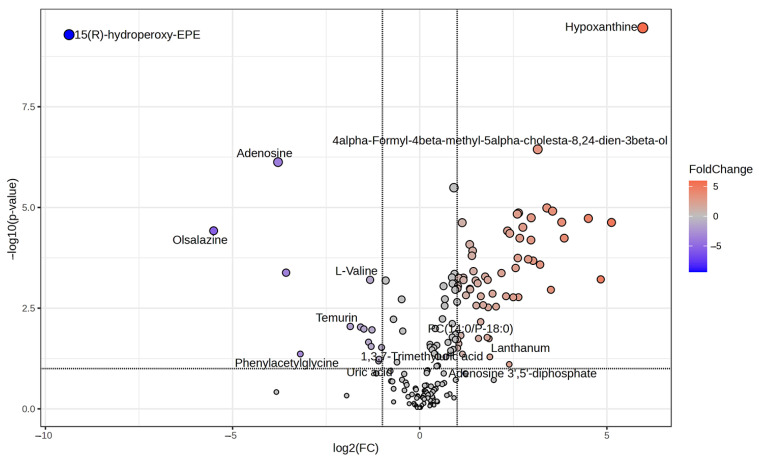
Volcano plot analysis of differential metabolites in drug-resistant SW620 cells. The x-axis represents log2 (fold change), while the y-axis represents the *p*-value in −log10 scale. The significantly upregulated metabolites are indicated in orange polka dots and those downregulated in blue polka dots (*p* < 0.05 and fold change > 1.5 or <0.75).

**Figure 5:**
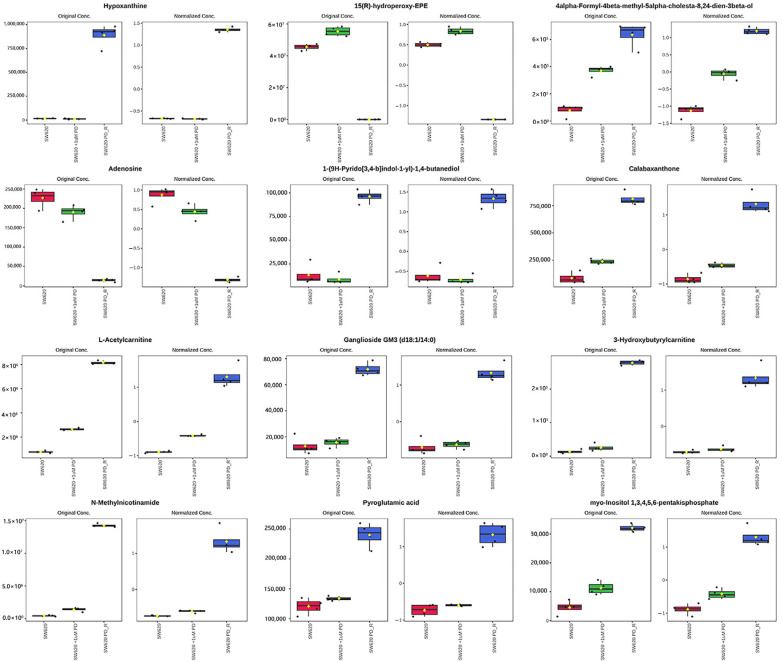
Significantly altered metabolites among the control and the palbociclib-treated groups. The box and whisker plots on the left show the original peak intensity values (mean ± SD). The box and whisker plots on the right summarize the normalized values. Red represents the expression level of metabolites in SW620 cells, green represents the expression level of metabolites in SW620 + 1 μM PD cells, and blue represents the expression level of metabolites in SW620 PD_R cells.

**Figure 6:**
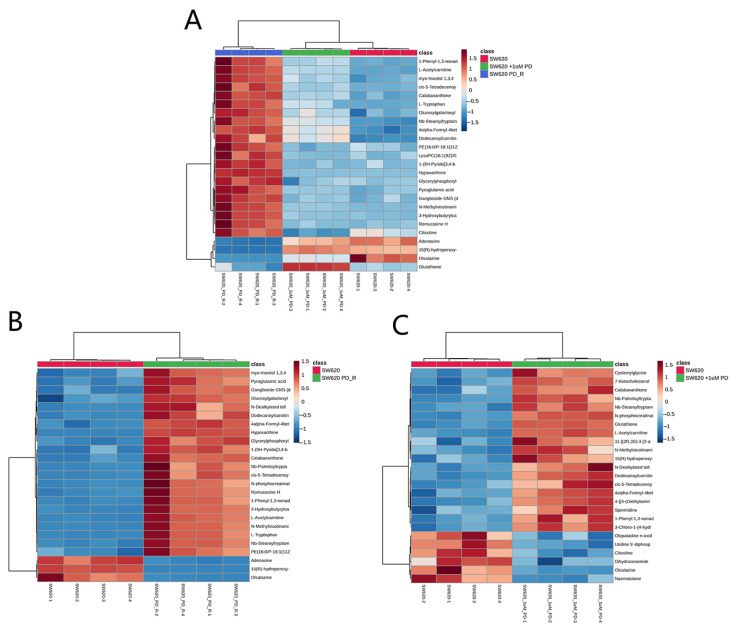
Hierarchical clustering heat map of the top 25 differential metabolites selected based on t-tests/ANOVA, with the degree of change marked with red (upregulation) and blue (downregulation). The distance measure was set to “Euclidean”, and the clustering algorithm was set to “Ward”. (**A**) Control group, SW620 + 1 μM PD group, and SW620 PD_R group. (**B**) Control group and SW620 PD_R group. (**C**) Control group and SW620 + 1 μM PD group.

As shown in the volcano plot (Figure 4), the right side of the valley plot represents the upregulated metabolites between the SW620 PD_R cell group and the SW620 cell group, while the left side of the valley plot represents the downregulated metabolites. The volcano plot for the SW620 + 1 μM PD cells and SW620 cells The volcano plots comparing the SW620 + 1 μM PD cells versus SW620 cells and SW620 PD_R cells versus SW620 + 1 μM PD cells are shown in Supplementary Figure S1, panels A and B, respectively. A bar chart count of the number of metabolites whose expression were up- or downregulated is shown in Supplementary Figure S2, where the number of significant changes in metabolic abundance in the SW620 PD_R group was significantly greater than that in the SW620 + 1 μM PD group. This suggests that the resistance developed in SW620 cells by prolonged exposure to pabocinib produces more significant interference with cellular metabolism. The analysis of variance (ANOVA) test was used to identify the potential biomarkers that contributed the most to the difference between the control group and the palbociclib treatment groups. The results with metabolites (*p* < 0.05) are shown in Table 1 and Figure 5 separately, which showed a high number of metabolites with differential expression and significant differences in expression in SW620 PD_R cells. It is suggested that sustained exposure to palbociclib exerts a pronounced influence on cellular metabolism, highlighting its potential impact on cellular homeostasis. A heatmap using the red and blue scale showed the first 25 metabolites that changed significantly (from high to low metabolic levels) (Figure 6). In pairwise comparisons, metabolites with a *p*-value below 0.05 and fold change above 1.5 or below 0.75 were selected as potential biomarkers. As shown in Supplementary Table S1, we summarized the identified metabolites and selected 11 metabolites as the marker metabolites that may have been altered in the SW620 PD_R cells compared to the control group. In addition, the disordered metabolites of *p* < 0.05 in the SW620 PD_R cells compared to the control group are summarized in Table 2. The corresponding results between the SW620 + 1 μM PD experimental group and the control group are shown in Supplementary Tables S2 and S3, respectively.

#### 3.2.2. Analysis of Metabolic Pathways

**Figure 7:**
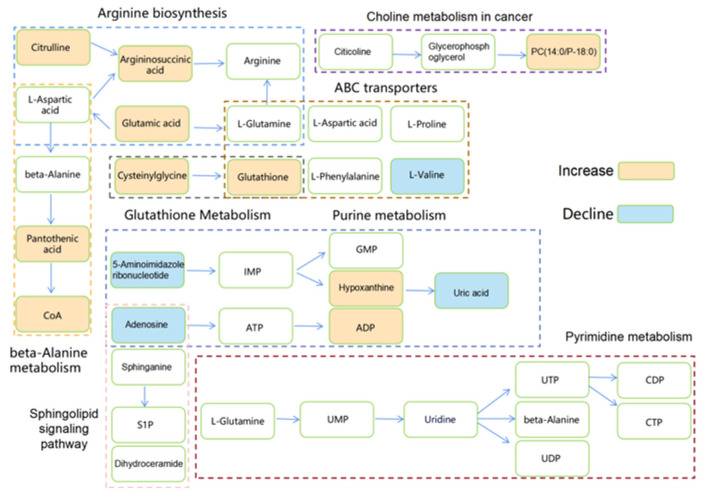
Schematic diagram of regulated metabolites and potential dysregulated metabolic pathways. Detected upregulated metabolites are shown with an orange background; downregulated metabolites are shown with a blue background; a blank background indicates no statistical significance or undetected. Abbreviations: CoA, coenzyme A; IMP, hypoxanthine; GMP, guanosine monophosphate; ADP, adenosine 3′,5′-diphosphate; UMP, uridine 5′-monophosphate; UTP, uridine triphosphate; UDP, uridine 5′-diphosphate; CDP, cytidine diphosphate; CTP, cytidine triphosphate.

We performed a metabolic pathway analysis of the metabolites in the SW620 PD_R cells using MetaboAnalyst 6.0 and Wukong Online. Wukong provides metabolic pathways from multiple pathway sources including KEGG, SMPDB, HumanCyc, etc., and we selected metabolic pathways from the KEGG pathway sources for analysis. Compared to the control group, several important drug resistant metabolic pathways were affected in the SW620 PD_R experimental group including purine metabolism, ABC transporters, pyrimidine metabolism, sphingolipid signaling pathway, beta-alanine metabolism, glutathione metabolism, arginine biosynthesis, and choline metabolism in cancer (Supplementary Table S4). Compared to the control group, several important drug resistant metabolic pathways were affected in the SW620 + 1 μM PD experimental group including beta-alanine metabolism, glutathione metabolism, inositol phosphate metabolism, glycerophospholipid metabolism, arginine and proline metabolism, and tryptophan metabolism (Supplementary Table S5). The metabolites with upregulated and downregulated expression are marked in orange and blue, respectively (Figure 7).

## 4. Discussion

Palbociclib is a commonly used chemotherapy drug, but cancer cells can become resistant to palbociclib [28,29]. In such scenarios, an escalation of palbociclib dosage becomes imperative to achieve therapeutic efficacy against cancer cells. Our findings showed that resistant SW620 PD_R cells exhibited higher IC50 values compared to the control group, indicating that only higher concentrations of palbociclib can effectively target these resistant cells. In order to explore the effect of palbociclib in drug-resistant cells and common cancer cells, drug-resistant cells SW620 PD_R and SW620 + 1 μM PD treated with 1 μM palbociclib were selected for follow-up experiments. Notably, palbociclib’s cytotoxic effect on cancer cells is accompanied by hematotoxicity, and high doses can impose a significant burden on patients. Studies have shown strict intake requirements for Palbociclib, and the starting intake of palbociclib is 125 mg per day for 3 weeks, followed by a one-week discontinuation [30].

Cyclin-dependent kinases (CDKs), a class of serine/threonine protein kinases, play a pivotal role in cell cycle regulation. Their enzymatic activity relies on the cyclin to provide the necessary domain, and its activity is affected by a combination of many signaling pathways and CDK inhibitors [31]. Aberrations in CDK regulation, such as the deletion of CDK inhibitors or the overexpression of CDKs or cyclins, can lead to cell cycle dysregulation and unchecked proliferation, which are hallmarks of malignancy [32,33]. The inhibition of CDK activity has become one of the methods for the treatment of malignant tumors. CDK4/6 is a key regulator of the cell cycle and is required for the development and progression of a variety of malignancies [34,35]. Palbociclib is a highly selective CDK4/6 inhibitory drug that regulates the cell cycle by blocking Rb dephosphorylation, preventing cells from the G1 phase to S phase and inhibiting DNA synthesis [30]. However, the emergence of resistance to CDK4/6 inhibitors in malignant tumors poses a challenge to their therapeutic efficacy and clinical application. The resistance mechanisms of CDK4/6 inhibitors can be broadly divided into cell cycle-specific mechanisms and non-specific mechanisms [36]. Mechanisms of cell cycle-specific resistance include Rb, E2F amplification, and the overexpression of CDK4 (INK4) family inhibitors [36,37,38]. The non-specific mechanisms of the cell cycle include the fibroblast growth factor receptor (FGFR1) pathway, the activation of the PI3K/Akt/mTOR signaling pathway, and the mitogen-activated protein kinase (MAPK) signaling pathway [39,40,41].

In this study, we further revealed the resistance mechanism of palbociclib to SW620 cells by the metabolomic approach. Using this approach, we found that 11 of the 76 differential metabolites (15 downregulated/61 upregulated) in the resistant SW620 PD_R cell experimental group compared to the control group (Table 2) showed significant changes (fold change > 1.5 or <0.75) (Supplementary Table S2). Pathways such as arginine biosynthesis were disrupted in the SW620 PD_R cell set while glutathione and beta-alanine metabolism and so on were significantly affected in the SW620 + 1 μM PD group.

The metabolism of inositol phosphates involves a series of phosphorylation and dephosphorylation reactions related to inositol. Inositol, a type of sugar found in various foods, exhibits multiple physiological functions within cells including participating in the composition of cell membranes, signal transduction, and energy metabolism. Phosphorylated inositol, such as inositol hexakisphosphate (IP6), possesses enhanced biological activity and can influence processes such as cell growth, differentiation, and apoptosis. Numerous studies have indicated that IP6 exhibits anticancer effects. In a study on the liver metastasis of colorectal cancer in mice, it was found that both IP6 and inositol exerted anticancer effects both in vitro and in vivo [42]. In the SW620 + 1 μM PD experimental group, the expression of IP6 decreased, which weakened its anticancer effect and facilitated the development of cancer cells and the emergence of drug resistance.

Therefore, these changes in the metabolic pathways reveal the metabolic impact of short-term exposure to palbociclib on SW620 cells, suggesting that during the initial stages of drug treatment, cells undergo rapid metabolic adaptation or stress responses. These responses are the subsequent occurrence of drug resistance. Below are the more specific metabolite analysis results from SW620 PD_R.

### 4.1. Amino Acid Metabolism

There are two arginine biosynthetic pathways involved in SW620 PD_R cells. One is where glutamate is converted into L-aspartate through transamination. L-aspartate and citrulline are the essential precursors for the synthesis of argininosuccinate, which is catalyzed by argininosuccinate synthetase to produce argininosuccinate [43]. Argininosuccinate is further cleaved to generate arginine [44]. The other pathway is where glutamate reacts with ammonia to produce L-glutamine, which is then indirectly converted into arginine through complex biochemical pathways [45]. The expression of argininosuccinic acid, citrulline, and glutamic acid was upregulated in the palbociclib-resistant SW620 PD_R cells, indicating that arginine metabolism was disturbed. Arginine plays an active role in tumor development and has a pro-tumor effect. High levels of arginine lead to the upregulation of ASNS expression in tumor cells, and the upregulation of ASNS expression can further enhance the ability of cells to uptake arginine, creating a positive feedback loop [46]. This also promotes the development of tumors from another perspective—immune cells need arginine to maintain anti-tumor function [47], and tumor cells consume a large amount of arginine in the tumor microenvironment, which helps them to escape immunosurveillance. A study on the microenvironment of liver cancer found that high levels of arginine in liver cancer cells were able to control gene expression and perform metabolic reprogramming after binding to RNA-binding motif protein 39 (RBM39), giving tumor cells the ability to divide indefinitely [46]. In the context of SW620 PD_R cells that demonstrated resistance to palbociclib, there was a conspicuous upregulation in the expression levels of arginine. This perturbation in arginine expression level translates to a disrupted metabolic homeostasis, which may ultimately pose a risk of facilitating malignant tumor progression and further fostering drug resistance.

In this study, metabolites in the SW620 PD_R cells participated in β-alanine metabolism, which encompasses the metabolic pathway of L-aspartic acid, β-alanine, pantothenic acid, and coenzyme A. Aspartic acid serves as the synthetic precursor of β-alanine, which is generated by the decarboxylation of aspartic acid [48]. β-Alanine then combines with hydroxybutyric acid to form pantothenic acid, and pantothenic acid is a crucial precursor for the synthesis of coenzyme A [49]. Vitamins are a class of trace organic substances that must be obtained from food in order to maintain normal physiological functions in humans and animals, and play an important role in human growth, development and metabolism. Vitamins are neither constituents of cells in living organisms nor do they provide energy to living organisms, but rather play a regulatory role in living organisms [50,51]. Pantothenic acid, also known as vitamin B5, plays a vital role in organisms. It maintains the health of the immune system and promotes cell composition and growth. In addition, pantothenic acid is also involved in energy metabolism. Pantothenic acid participates in the synthesis of coenzyme A, which serves as a versatile and essential cofactor in a variety of metabolic reactions including the tricarboxylic acid cycle [52], fatty acid synthesis and degradation [53,54], etc. Pantothenic acid and coenzyme A play an important role in tumor development. In human cancers, there exists a proto-oncogene named MYC, which can affect the expression of several genes and regulate cell proliferation under normal conditions. However, during tumor progression, MYC can be mutated or overexpressed, promoting tumor development [55]. In a study of breast tumor cells, based on the fact that pantothenic acid is a precursor for the synthesis of coenzyme A, it was found that an increase in MYC activity could help tumors elevate the uptake and metabolism of pantothenic acid, promote the tricarboxylic acid cycle, and stimulate high rates of tumor growth [56]. It has been shown that overexpression of the MYC gene in colorectal cancer promotes the malignant development of tumors [57]. In the experimental group of drug-resistant SW620 PD_R cells, the expression of coenzyme A was upregulated, so it is possible that the overexpression of MYC increased the uptake of pantothenic acid by the tumors that promoted the synthesis of coenzyme A, and then promoted the tricarboxylic acid cycle, which helped in the rapid growth of the tumors and increased their resistance.

### 4.2. Glutathione Metabolism

In this study, the SW620 PD_R experimental group with palbociclib resistance was upregulated in cysteinylglycine expression compared to the control group. This elevation suggests an upregulation in glutathione expression, as cysteinylglycine is a dipeptide composed of cysteine and glycine, constituting a structural component of glutathione. In the study of drug resistance to neuroblastoma, the expression of glutathione in cells is upregulated [58]. The amount of glutathione expressed in cancer cells plays a crucial role in regulating the mutagenesis mechanism and tumor resistance to drugs [59]. Several studies have shown that high levels of glutathione not only promote the growth and development of tumors, but also lead to the emergence of drug resistance in cancer [58,60,61]. Therefore, the increased expression of glutathione may be one of the reasons for the resistance of SW620 cells to palbociclib.

### 4.3. ABC Transporters

ABC transporters are a class of membrane proteins widely present in organisms. Given that they all contain adenosine triphosphate (ATP) binding cassettes within their structures and can utilize the energy released from ATP hydrolysis to transport substances, they are therefore referred to as ATP-binding cassette transporters (ABC transporters). ABC transporters facilitate the transmembrane transport of various substrates including ions, metabolites, drugs, and more by harnessing the energy generated from ATP hydrolysis, thus participating in numerous vital physiological processes [62].

ABC transporters play a pivotal role in the drug resistance of cancer cells due to their broad substrate spectrum, enabling them to recognize and transport a wide range of anticancer drugs. By mediating the efflux of these drugs, these transporters reduce the effective intracellular drug concentration, thereby weakening therapeutic efficacy and potentially leading to treatment failure. Numerous studies have investigated the contribution of ABC transporters to cancer cell resistance. P-glycoprotein, encoded by the MDR1/ABCB1 gene, is one of the major factors contributing to the drug-resistant phenotype of cells. This protein modulates the ATP-dependent efflux of anticancer drugs, thereby conferring resistance to cancer cells [63]. In a study focusing on the resistance of human ovarian cancer cells, researchers examined the role of dual-specificity phosphatase 1 (DUSP1) in the development of resistance to paclitaxel in these cells. They found that the overexpression of DUSP1 upregulated P-glycoprotein expression, which facilitated the efflux of anticancer drugs, thereby leading to resistance in human ovarian cancer cells [64]. Furthermore, in the context of non-small cell lung cancer (NSCLC) cells, researchers investigated the relationship between Slug, a transcription factor that induces epithelial-mesenchymal transition (EMT), and MRP2, an efflux transporter. Their findings revealed that Slug enhanced MRP2 expression, which facilitated drug efflux, conferring resistance to NSCLC cells [65]

Glutathione, as an important intracellular antioxidant and detoxifier, can directly participate in the metabolism and excretion of various drugs and toxins. It binds to drugs and their metabolites through the sulfhydryl group (-SH) on its cysteine, forming conjugates that reduce the toxicity of these substances and facilitate their elimination [66]. ABC transporters, as essential transporters on the cell membrane, actively transport drugs and their metabolites from inside the cell to the outside. In some cases, glutathione also serves as a co-transport substrate or cofactor, synergizing with ABC transporters to promote the transmembrane transport of drugs [67].

In this study, the pathways related to the metabolites of the SW620 PD_R drug-resistant cell line were associated with glutathione and ABC protein transport. This is based on the synergistic transport effect of ABC transporters and glutathione, which enables the efflux of various anticancer drugs across membranes out of cells, thereby conferring drug resistance to the cells. This is a crucial point in the research of anti-cell drug resistance.

### 4.4. Purine Metabolism

Purine metabolism provides cells with the necessary energy and cofactors to promote cell survival and proliferation, and purine metabolism is associated with the development of cancer [68]. Cell division and tumor cell proliferation have a high demand for purine nucleotides, and in humans, the synthesis of purines relies on purine enzymes to catalyze the conversion of phosphoribosyl pyrophosphate to adenosine [69]. Cancer drug resistance is the main obstacle to cancer treatment, and purine metabolism disorders are closely related to cancer drug resistance. Purine signaling regulates the tumor microenvironment, epithelial–mesenchymal transition, and anti-tumor immunity, thereby affecting tumor sensitivity to drugs [70]. In one study, the purine metabolite levels were found to be significantly elevated in oxaliplatin-resistant colorectal cancer [71]. In SW620 PD_R cells, the expression of adenosine 3′,5′-diphosphate and 5-aminoimidazole ribonucleotide related to purine metabolism was upregulated, and purine metabolism was increased, creating a suitable microenvironment for tumors, promoting the proliferation of tumor cells, and reducing the sensitivity of tumor cells to drugs.

### 4.5. Sphingolipid Signaling Pathway

The sphingolipid signaling pathway is closely related to drug resistance in cancer. In this study, the metabolites adenosine, sphingosine, and dihydroceramide from SW620 PD_R cells participated in the sphingolipid signaling pathway. Adenosine, which is produced during the catabolism of ATP, is involved in energy metabolism, providing conditions for the rapid proliferation of cancer cells [72]. Meanwhile, it also supplies energy support for efflux pumps that can expel drugs out of the cells [73]. Adenosine is involved in the immune regulation of cancer by inhibiting the functions of various immune cells including NK cells and T cells through the activation of receptors such as A2AR [74,75,76]. The immunosuppressive effect of adenosine helps cancer cells evade the surveillance and attack of the immune system, enabling their massive proliferation. Additionally, adenosine can act on vascular endothelial cells, promoting the formation of new blood vessels in tumors, thereby providing necessary nutrients and oxygen for tumor growth and metastasis [77].

Sphingosine is a crucial intermediate product in sphingolipid metabolism, playing a significant role in cellular processes such as growth, proliferation, and migration. In sphingolipid metabolism, sphingosine kinase catalyzes the conversion of sphingosine into sphingosine-1-phosphate (S1P) [78]. S1P acts as a messenger molecule that promotes growth and inhibits apoptosis, activating various signaling pathways like PI3K/Akt, thereby facilitating the proliferation and survival of tumor cells and contributing to their resistance to chemotherapeutic drugs [79]. Additionally, S1P promotes the expression and secretion of vascular endothelial growth factor (VEGF), enhances the migration and proliferation of angiogenesis-related cells, and thus participates in the formation of new blood vessels in tumors, playing a pivotal role in tumor growth and metastasis [80]. In conclusion, the sphingolipid signaling pathway is closely related to the development of drug resistance in SW620 cells.

### 4.6. Choline Metabolism in Cancer

Choline metabolism involves multiple enzymatic reactions and signaling pathways. In cancer cells, choline metabolism often undergoes abnormalities, which include changes in the expression of metabolic enzymes, the accumulation of metabolic products, or the reprogramming of metabolic pathways. These abnormal changes not only provide necessary materials and energy support for cancer cell growth and proliferation, but also participate in cancer progression and the formation of drug resistance through mechanisms such as affecting cell signal transduction and epigenetic regulation [81,82]. In SW620 PD_R cells, choline metabolism involves cytidine diphosphate-choline (CDP-choline), phosphatidylcholine, and PC(14:0/P-18:0). In organisms, CDP-choline can be enzymatically converted into phosphatidylcholine [83], which in turn can be degraded by phospholipases to generate glycerophosphocholine. Phosphatidylcholine interacts with drug transporter proteins such as P-glycoprotein (P-gp) and multidrug resistance-associated protein (MRP). The transporter proteins can pump drugs out of cells, thereby reducing the efficacy of drugs and producing drug resistance [84]. Therefore, phosphatidylcholine may be one of the factors contributing to the drug resistance of SW620 cells.

### 4.7. Pyrimidine Metabolism

Pyrimidine is an important component of nucleic acids (DNA and RNA), and pyrimidine metabolism plays a crucial role in the process of cell proliferation and differentiation. In cancer cells, due to the accelerated cell proliferation rate, the demand for pyrimidine also increases correspondingly. The metabolites in SW620 PD_R cells involve pyrimidine metabolism, where L-glutamine generates uridine-5′-monophosphate (UMP). Uridine is phosphorylated to produce uridine-5′-monophosphate [85]. Uridine can also generate uridine diphosphate (UDP) and uridine triphosphate (UTP) [80]. Among them, UTP can be further decomposed into cytidine diphosphate (CDP) and cytidine triphosphate (CTP) [86].

Uridine monophosphate (UMP), uridine diphosphate (UDP), and uridine triphosphate (UTP), as crucial intermediates in the metabolic process of pyrimidine nucleotides, exhibit intricate relationships with drug resistance in cancer cells. Studies have revealed that the pathway enzyme cytidine deaminase (CDA) in cancer cells contributes to the production of uridine diphosphate (UDP). Extracellular UDP, through its receptor P2Y6 on macrophages, disrupts normal cellular functions, thereby promoting immunosuppression and resistance to immunotherapy [87]. In a study on drug resistance factors of esophageal squamous cell carcinoma, it was found that the expression of UTP3 in drug-resistant cancer cells was significantly higher than that in drug-sensitive cells. UTP3 recruits c-Myc to activate the expression of vesicle-associated membrane protein 3 (VAMP3). Activated VAMP3 inhibits caspase-dependent apoptosis and ultimately leads to chemotherapy resistance [88].

The oncogene MYC supports cancer cell proliferation and survival by concurrently inducing multiple anabolic processes. Studies have found that the inhibition of CTP synthase (CTPS) reduces cellular activity, and MYC-driven rRNA synthesis causes DNA replication stress when CTPS is inhibited. Combined inhibition of CTPS and ataxia telangiectasia and Rad3-related protein (ATR) exhibits synthetic lethality in MYC-overexpressing cells, promoting cell death in vitro and reducing tumor growth in vivo. This suggests that CTP can promote cancer cell proliferation, thereby making cancer cells resistant to drugs [89].

Therefore, based on the close relationship between pyrimidine metabolism and the drug resistance of cancer cells, it may be considered as a factor contributing to the drug resistance of SW620 cells.

### 4.8. Limitations

#### 4.8.1. Limitations of LC-MS

Despite the crucial role Liquid Chromatography-Mass Spectrometry (LC-MS) plays as a powerful analytical tool in metabolomics research, providing high sensitivity and high-resolution metabolite detection capabilities, we must recognize that LC-MS technology itself has certain limitations.

Firstly, LC-MS does not cover all metabolites. Metabolites are diverse in species and chemical properties, and the detection capabilities of LC-MS are limited by its separation and detection principles. Some metabolites may be difficult to effectively detect by LC-MS due to their instability under specific conditions, polarity, or molecular structural characteristics. Therefore, when interpreting LC-MS data, we need to carefully consider its detection range to avoid over-interpretation or missing important information.

Secondly, technical limitations may also result in some metabolites not being detected. For example, losses during sample processing, differences in instrument performance, and limitations in data analysis methods may all affect the detection effectiveness of metabolites. To minimize the impact of these technical limitations on research results, we have adopted strict quality control measures in experimental design and data analysis processes. During the research process, we have optimized experimental conditions as much as possible, selected appropriate metabolite extraction and enrichment methods, and employed multiple data processing and analysis strategies to reduce potential technical biases and omissions.

In summary, although LC-MS has significant advantages in metabolomics research, we also need to face its limitations and take corresponding measures in experimental design and data analysis processes to ensure the accuracy and reliability of research results. In the future, with the continuous advancement of technology and the continuous improvement of methods, we are confident that we can more comprehensively reveal changes in metabolites, providing stronger support for a deeper understanding of biological processes and disease mechanisms.

#### 4.8.2. Limitations of the Experiment and Future Directions for the Experiment

Although our study provides valuable insights into the metabolic changes in palbociclib-resistant colorectal cancer cells, we must acknowledge several limitations. Firstly, our work lacked subsequent experiments, such as pathway inhibition experiments or orthogonal experiments, to further validate the functional role of the identified metabolic pathways in conferring drug resistance. These experiments could provide us with a deeper understanding of the mechanisms of palbociclib resistance and potentially discover novel therapeutic targets to overcome this resistance.

Secondly, we did not investigate whether the metabolic changes observed after the cessation of palbociclib exposure were reversible. Understanding whether these changes are reversible or persistent would provide us with valuable information regarding the durability of the resistance mechanisms and their potential impact on long-term treatment outcomes. Such studies may have significant implications for the clinical management of patients with palbociclib-resistant colorectal cancer.

Given these limitations, we recognize the need for further research to fill these gaps. Future studies should include pathway inhibition experiments and orthogonal approaches to validate the functional contribution of the identified metabolic pathways. Additionally, investigating the reversibility of metabolic changes after drug withdrawal will provide us with a more comprehensive understanding of the resistance mechanisms and their durability. We hope that our current findings can lay the foundation for these future research efforts and contribute to the ongoing efforts to overcome drug resistance in cancer treatment.

## 5. Conclusions

In this study, LC-MS metabolomics was used to investigate the metabolite changes of SW620 cells that were resistant to palbociclib. Research data showed that the altered metabolites included amino acids, glutathione, and so on, especially related to arginine biosynthesis, purine metabolism, and ABC transporters. These results are helpful to understand the metabolic processes of SW620 cells that are resistant to palbociclib, and may provide potential clues for discovering the potential resistance mechanism caused by palbociclib as well as data to further advance the research on anti-palbociclib resistance.

## Figures and Tables

**Table 1:** Significantly altered metabolites among the control and the Plabociclib-treated groups by ANOVA test analysis with Tukey’s post hoc analysis.

	f.Value	*p*.Value	Fisher’s LSD
Hypoxanthine	5578.3	1.20 × 10^−14^	SW620 PD_R − SW620; SW620 PD_R − SW620 + 1 μM PD
15(R)-hydroperoxy-EPE	1478.5	4.67 × 10^−12^	SW620 + 1 μM PD − SW620; SW620 − SW620 PD_R; SW620 + 1 μM PD − SW620 PD_R
4alpha-Formyl-4beta-methyl-5alpha-cholesta-8,24-dien-3beta-ol	274.17	8.64 × 10^−9^	SW620 + 1 μM PD − SW620; SW620 PD_R − SW620; SW620 PD_R − SW620 + 1 μM PD
Adenosine	201.52	3.36 × 10^−8^	SW620 − SW620 + 1 μM PD; SW620 − SW620 PD_R; SW620 + 1 μM PD − SW620 PD_R
1-(9H-Pyrido[3,4-b]indol-1-yl)-1,4-butanediol	154.04	1.09 × 10^−7^	SW620 PD_R − SW620; SW620 PD_R − SW620 + 1 μM PD
Calabaxanthone	153.97	1.10 × 10^−7^	SW620 + 1 μM PD − SW620; SW620 PD_R − SW620; SW620 PD_R − SW620 + 1 μM PD
L-Acetylcarnitine	145.74	1.39 × 10^−7^	SW620 + 1 μM PD − SW620; SW620 PD_R − SW620; SW620 PD_R − SW620 + 1 μM PD
Ganglioside GM3 (d18:1/14:0)	144.72	1.44 × 10^−7^	SW620 PD_R − SW620; SW620 PD_R − SW620 + 1 μM PD
3-Hydroxybutyrylcarnitine	140.57	1.63 × 10^−7^	SW620 PD_R − SW620; SW620 PD_R − SW620 + 1 μM PD
N-Methylnicotinamide	131.68	2.17 × 10^−7^	SW620 PD_R − SW620; SW620 PD_R − SW620 + 1 μM PD
Pyroglutamic acid	130.87	2.23 × 10^−7^	SW620 PD_R − SW620; SW620 PD_R − SW620 + 1 μM PD
myo-Inositol 1,3,4,5,6-pentakisphosphate	122.13	3.01 × 10^−7^	SW620 + 1 μM PD − SW620; SW620 PD_R − SW620; SW620 PD_R − SW620 + 1 μM PD

**Table 2:** The disturbed metabolites with *p* < 0.05 in drug-resistant cells SW620 cells compared to controls.

	Raw. *p*Val	FC
Hypoxanthine	3.48 × 10^−10^	62.146
15(R)-hydroperoxy-EPE	5.14 × 10^−10^	0.0015131
4alpha-Formyl-4beta-methyl-5alpha-cholesta-8,24-dien-3beta-ol	3.61 × 10^−7^	8.8911
Adenosine	7.49 × 10^−7^	0.072479
Calabaxanthone	1.03 × 10^−5^	10.54
L-Acetylcarnitine	1.23 × 10^−5^	11.702
Nb-Stearoyltryptamine	1.37 × 10^−5^	6.2214
Ganglioside GM3 (d18:1/14:0)	1.46 × 10^−5^	6.0916
1-(9H-Pyrido[3,4-b]indol-1-yl)-1,4-butanediol	1.80 × 10^−5^	7.8753
3-Hydroxybutyrylcarnitine	1.88 × 10^−5^	22.698
L-Tryptophan	2.32 × 10^−5^	13.867
N-Methylnicotinamide	2.36 × 10^−5^	34.831
Pyroglutamic acid	2.39 × 10^−5^	2.1938
myo-Inositol 1,3,4,5,6-pentakisphosphate	3.11 × 10^−5^	6.7494
Olsalazine	3.79 × 10^−5^	0.021995
Glucosylgalactosyl hydroxylysine	3.83 × 10^−5^	5.0824
Dodecanoylcarnitine	4.41 × 10^−5^	5.2966
1-Phenyl-1,3-nonadecanedione	5.78 × 10^−5^	14.523
cis-5-Tetradecenoylcarnitine	5.80 × 10^−5^	6.3735
N-Dealkylated tolterodine	6.45 × 10^−5^	7.8416
Romucosine H	8.28 × 10^−5^	2.5224
PE(16:0/P-18:1(11Z))	0.00011922	2.6627
N-phosphocreatinate(2-)	0.00015805	2.6268
Nb-Palmitoyltryptamine	0.00018002	6.1449
LysoPC(16:1(9Z)/0:0)	0.00019323	7.4486
Cycloartanyl ferulate	0.00021002	8.1567
7-Ketocholesterol	0.00026212	9.2833
LysoPE(P-16:0/0:0)	0.00031867	5.9015
2,5-Dihydro-2,4,5-trimethyloxazole	0.00038177	2.7032
2-(2,5-dihydroxy-4-methoxyphenyl)-3,5,6,7-tetrahydroxy-3,4-dihydro-2H-1-benzopyran-4-one	0.00041611	0.084164
D-myo-Inositol 1,4-bisphosphate	0.00042566	4.5423
Tripropylamine	0.0005206	3.3591
L-3-Aminodihydro-2(3H)-furanone	0.00055385	2.243
3-(Methylthio)propyl acetate	0.00056087	2.0604
LysoPE(P-18:1(9Z)/0:0)	0.00061073	28.554
L-Valine	0.00062722	0.39883
Eplerenone	0.00062787	3.5195
15-deoxy-delta12,14-Prostaglandin J2-2-glycerol ester	0.0006347	2.2486
Modafinil	0.00065158	2.8166
SM(d18:1/16:0)	0.00076249	2.9213
N1,N8-Diacetylspermidine	0.00089671	2.0289
[(7-hydroxy-1-oxo-1H-isochromen-3-yl)methoxy]sulfonic acid	0.0010282	2.015
N-Jasmonoylisoleucine	0.0010399	2.5317
1-(Hydroxymethyl)-5,5-dimethyl-2,4-imidazolidinedione	0.0010871	2.5453
Hexylcaine	0.0011073	11.345
PE(18:1(11Z)/P-18:1(11Z))	0.0013814	3.8516
Celecoxib	0.0015148	2.3525
Isoleucylproline	0.0016004	3.1001
Tetrahydrofuran	0.001601	4.9395
Spermidine	0.0016885	6.2252
Ketoprofen	0.0016981	5.6494
Isobutyryl-L-carnitine	0.0026473	3.2364
2-Propionyl-2-thiazoline	0.0027183	2.8454
S-Acetyl dihydroasparagusic acid	0.0028977	4.108
Coenzyme A	0.0030194	3.5508
PC(14:0/P-18:0)	0.0069042	3.0879
Temurin	0.0089961	0.27611
5-Aminoimidazole ribonucleotide	0.009336	0.33438
8-Hydroxypinoresinol 8-glucoside	0.010413	0.35534
(S1)-Methoxy-3-heptanethiol	0.010941	0.4128
Spermine	0.01511	2.1413
Glycerophosphoinositol	0.016882	3.4815
Methocarbamol	0.018028	3.6093
Cysteinylglycine	0.018969	2.0213
Dihydrolipoamide	0.022107	0.38748
(S)-3-Mercaptohexyl pentanoate	0.024091	2.0645
(Cyclohexylmethyl)pyrazine	0.028188	0.40684
Uridine diphosphate glucuronic acid	0.029799	0.49321
Citrulline	0.031124	2.0181
Phenylacetylglycine	0.04339	0.10952
Argininosuccinic acid	0.043418	2.2053

## Data Availability

The metabolomics data have been deposited in the public database MetaboLights and can be accessed by visiting the website at www.ebi.ac.uk/metabolights/MTBLS10375 (accessed on 4 June 2024) and searching for project number MTBLS10375.

## Supplementary Materials

The following supporting information can be downloaded at: https://www.mdpi.com/article/10.3390/cimb47010024/s1.
