# Supplementary material for: Elucidating the Mechanisms of Acquired Palbociclib Resistance via Comprehensive Metabolomics Profiling
Source: Curr Issues Mol Biol. 2025 Jan 2;47(1):24. doi: 10.3390/cimb47010024 (PMC11763656; doi:10.3390/cimb47010024)
Supplement: Supplementary file 1 [file cimb-47-00024-s001.zip › Supplemental Figure Legends.pdf]

Supplemental Figure Legends

Supplemental Figure S1 A: Differential volcano plot of metabolite expression in SW620+1 μM PD cells compared to SW620 cells. B: Differential volcano plot of metabolite expression in SW620 PD_R cells compared to SW620+1 μM PD cells. The x-axis represents log2 (fold change), while the y-axis represents p-value in -log10 scale. The significantly up-regulated metabolites were indicated in orange polka dots and down-regulated in blue polka dots. ($p < 0.05$ and fold change >1.5 or <0.75).

Supplemental Figure S2 Bar graph of the number of metabolites with altered expression in paboxinib-treated SW620 cells compared to untreated SW620 cells. Red bars: number of metabolites with up-regulated expression; blue bars: number of metabolites with down-regulated expression.
