# Supplementary material for: Elucidating the Mechanisms of Acquired Palbociclib Resistance via Comprehensive Metabolomics Profiling
Source: Curr Issues Mol Biol. 2025 Jan 2;47(1):24. doi: 10.3390/cimb47010024 (PMC11763656; doi:10.3390/cimb47010024)
Supplement: Supplementary file 1 [file cimb-47-00024-s001.zip › Supplemental Table Legends.pdf]

Supplemental Table Legends

Supplemental Table S1 Marker metabolites that may be altered in resistant cells SW620 compared to controls $p<0.05, FC>1.5$ or <0.75.

Supplemental Table S2 Potential metabolic markers in SW620 cells treated with 1 μM Plabociclib compared to controls $p<0.05, FC<1.5$ or <0.75.

Supplemental Table S3 All disordered metabolites ($p<0.05$) in SW620+1 μM PD cells compared to controls.

Supplemental Table S4 Altered metabolic pathways in resistant SW620 cells compared to controls.

Supplemental Table S5 Altered metabolic pathways in SW620+1 μM PD cells compared to controls.
