# Supplementary figures and images for: Elucidating the Mechanisms of Acquired Palbociclib Resistance via Comprehensive Metabolomics Profiling
Source: Curr Issues Mol Biol. 2025 Jan 2;47(1):24. doi: 10.3390/cimb47010024 (PMC11763656; doi:10.3390/cimb47010024)

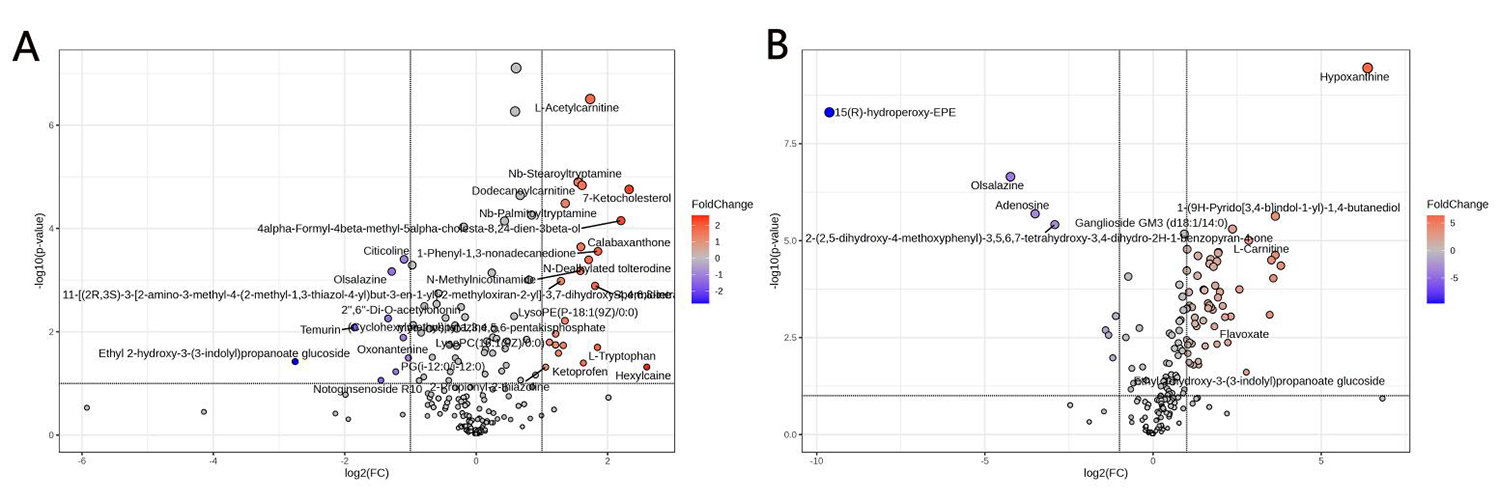

Supplement: Supplementary file 1 [file cimb-47-00024-s001.zip › Supplemental Figure S1.tif]

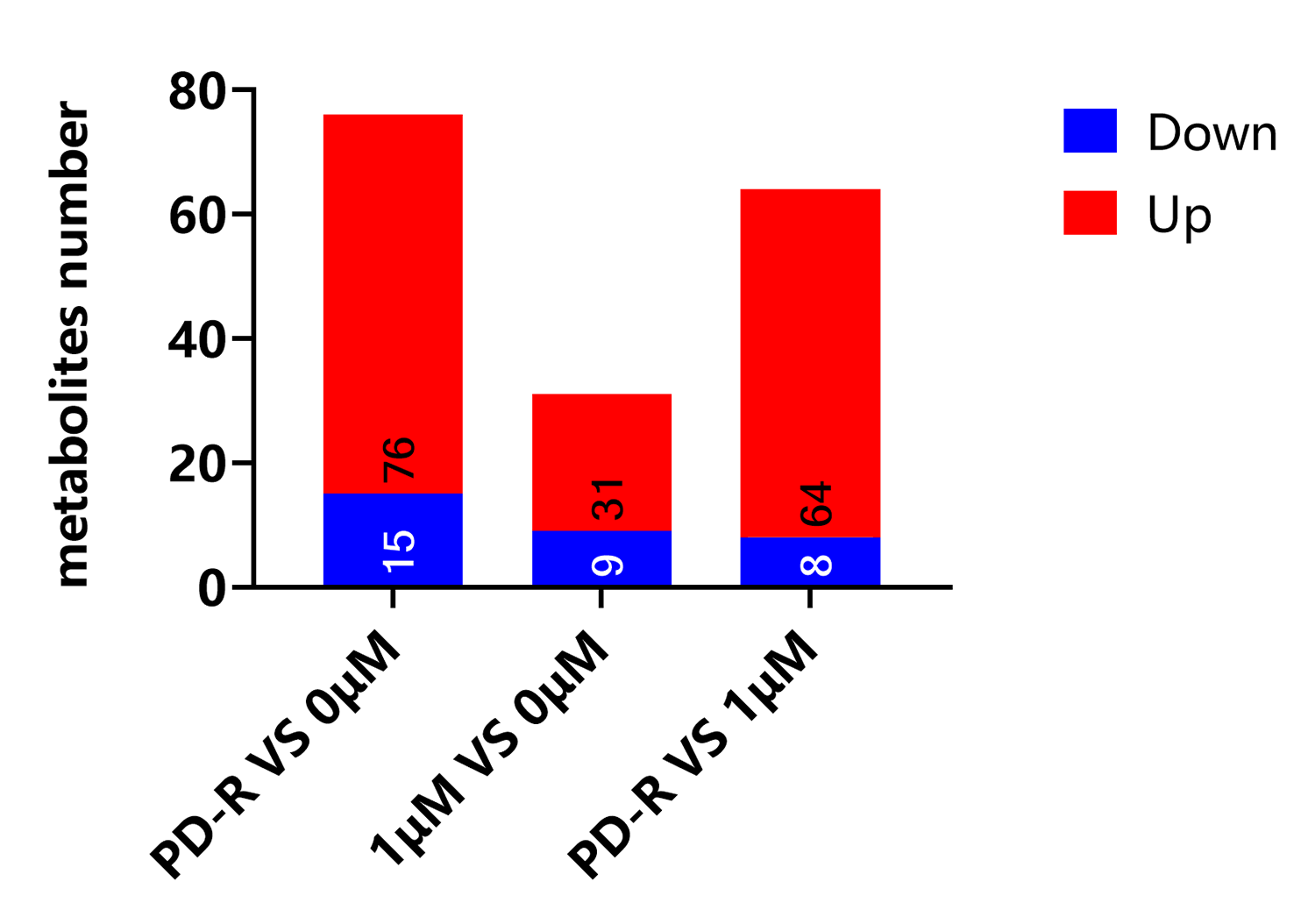

Supplement: Supplementary file 1 [file cimb-47-00024-s001.zip › Supplemental Figure S2.tif]
